# Distinct structural mechanisms drive gain-of-function activation of TMEM16E in gnathodiaphyseal dysplasia

**DOI:** 10.21203/rs.3.rs-10425456/v1

**Published:** 2026-09-15

**Authors:** Eleonora Di Zanni, Omar E. Alvarenga, Nicole Rychlik, Zhang Feng, Elizabeth D. Kim, George Khelashvili, Alessio Accardi

**Affiliations:** 1 Department of Anesthesiology, Weill Cornell Medicine; 2 Physiology, Biophysics, and Systems Biology Graduate Program, Weill Cornell Medicine; 3 ChemBion graduate program, University of Münster, Münster, Germany; 4 Institute of Physiology I, University of Münster, Münster, Germany; 5 Department of Computational and Systems Biomedicine, Weill Cornell Medicine; 6 Department of Biochemistry and Biophysics, Weill Cornell Medicine.

## Abstract

In cells TMEM16E (ANO5) mediates Ca^2+^-dependent currents and lipid scrambling. Its mutations cause muscular dystrophies and gnathodiaphyseal dysplasia 1 (GDD), but its activation mechanism is unknown. We show purified TMEM16E is a scramblase and determined cryoEM structures in apo and Ca^2+^-bound states. Unlike other TMEM16s, apo TMEM16E has a straight TM6 helix and preformed orthosteric sites; Ca^2+^ binding induces no rearrangements, and one site remains partially unoccupied in saturating Ca^2+^. Structures of two GDD gain-of-function mutants, G503E and R582I, show that despite similar functional phenotypes they act through distinct mechanisms: G503E disrupts the TM3–TM4 interface, while R582I remodels an extracellular loop network and increases S2 occupancy. Molecular dynamics simulations show G503E favors an X-shaped groove that scrambles lipids outside the groove, resembling active TMEM16F, rather than the open-groove mechanism of the ER-resident TMEM16K. Thus, phenotypically convergent mutations act through distinct pathways, paving the way for development of targeted therapies.

## Introduction

The TMEM16E protein (also known as ANO5) is a member of the TMEM16 family of Ca^2+^-activated ion channels and phospholipid scramblases ^1, 2, 3, 4^. The human genome contains 10 TMEM16 genes, and their mutations are associated with a variety of inheritable disorders of blood, bone, muscle and nervous system ^3, 5, 6^. Further, dysregulated activity of TMEM16 proteins is associated with a wide range of proliferative phenotypes ^7, 8, 9, 10, 11^. TMEM16E is highly expressed in muscle and bone ^12^, and was originally identified as the gene responsible for the autosomal dominant disorder gnathodiaphyseal dysplasia 1 (GDD) ^13, 14^, a disorder characterized by cemento-osseous lesions of the mandible and maxilla, bowing and sclerosis of long bones, and bone fragility leading to recurrent fractures ^15, 16, 17, 18, 19, 20, 21, 22, 23, 24, 25, 26, 27^. Homozygous and compound heterozygous mutations (missense or frameshift mutations) in TMEM16E are associated with autosomal recessive limb-girdle muscular dystrophy-12 (LGMDR12; MIM# 611307) and Miyoshi muscular dystrophy-3 (MMD3; MIM# 613319) ^28, 29^. Notably, both phenotypes can coexist in some patients ^30^. In animal models, defective TMEM16E leads to dysregulated Ca^2+^ homeostasis ^31, 32^, reduced fertility and sperm motility ^33^, abnormal sarcolemma repair, defective myoblast regeneration and fusion ^34, 35, 36^. Finally, dysregulation of the TMEM16E gene is associated with multiple neoplasia, where its upregulation promotes cell proliferation and migration, osteosarcoma ^37^, gastric ^10^ and pancreatic ^7^ cancers, while its downregulation promotes thyroid cancer ^37, 38^. Together, these findings highlight the importance of TMEM16E in human patho-physiology.

Despite its importance, our understanding of TMEM16E function and the mechanisms underlying its dysfunction in disease remain poorly understood. At rest, TMEM16E is primarily localized to the endoplasmic reticulum (ER), which has hindered its functional characterization ^33^. In muscle cells, activation of the damage repair cascade results in trafficking of TMEM16E to the plasma membrane (PM), where it facilitates progenitor cell fusion and membrane repair ^34, 35, 36^. Overexpression studies showed TMEM16E, like other TMEM16 family members ^39, 40, 41^, is likely a dual-function Ca^2+^-activated phospholipid scramblase and non-selective ion channel with an EC50 ~3 μM ^19, 21^. Notably, GDD TMEM16E mutants have a gain of function (GoF) phenotype with constitutive scramblase and ion channel activity at basal cellular Ca^2+^ levels ^19, 21^. In contrast, variants associated with muscular dystrophies are generally nonsense or loss-of-function (LoF) mutations ^28, 29^, suggesting the disease phenotypes correlate with the functional effects on TMEM16E, further underscoring the importance of understanding its activation and regulation mechanisms.

The Ca^2+^-activated TMEM16 proteins are double-barreled homodimers (Supplementary Fig. 1a,c), where each protomer forms independent hydrophilic grooves that serve as permeation pathways for ions and/or lipids that are delimited by TM3-TM6 (Supplementary Fig. 1b,d) ^2, 39, 42, 43, 44, 45, 46, 47^. Whereas TMEM16A and B are chloride channels, most other family members are dual function non-selective ion channels and lipid scramblases ^1, 2, 3, 4^. TMEM16 activation is triggered by Ca^2+^ binding to two sites located near the permeation pathway (Supplementary Fig. 1e), called orthosteric sites S1 and S2, which are formed by residues on TM6-TM8. In mammalian homologues, a third binding site located near the dimer interface has been proposed to play a regulatory role ^39, 48^, termed S3 allosteric site (Supplementary Fig. 1f). In all TMEM16s, in the absence of Ca^2+^, TM6 is bent disrupting the formation of the orthosteric sites, and the groove is closed by juxtaposition of TM4 and TM6. Ca^2+^ binding induces a straightening of TM6 to form the orthosteric sites ^2, 39, 42, 43, 44, 45, 46, 47^. Despite structural and functional conservation, divergent activation mechanisms have been proposed for different TMEM16 scramblases. In the ER-resident TMEM16K and in the fungal afTMEM16 and nhTMEM16, Ca^2+^ binding to S1 and S2 induces a rearrangement where TM4 detaches from TM6, opening the hydrophilic groove to the membrane core and forming a shared pathway for ions and lipids (Supplementary Fig. 1b) ^2, 39, 42, 43^. In contrast, activation of TMEM16F entails a rearrangement where TM4 slides along TM6 to generate an X-shaped groove that forms separate pathways for ions and lipids (Supplementary Fig. 1c,d) ^49, 50^. Notably, it has been proposed that some gain of function mutations could lead to groove opening in TMEM16F ^51, 52^. The Ca^2+^-dependent activation mechanism of TMEM16E is unknown, as is the mode of action of the GoF and LoF mutations associated with disease phenotypes.

Here, we addressed these questions by purifying human TMEM16E (hTMEM16E), reconstituting it in liposomes and showing it functions as a Ca^2+^-activated lipid scramblase, although its ligand-dependence is lower compared to other family members. We used cryogenic electron microscopy (cryoEM) to determine its structure in the apo and Ca^2+^-bound states and found that in apo hTMEM16E the TM6 helix is straight, the orthosteric sites are pre-formed, Ca^2+^ binding does not induce any rearrangements in the transmembrane region, and the S2 orthosteric site is not fully occupied, even in millimolar Ca^2+^. Consistently, mutations disrupting the orthosteric sites do not completely ablate Ca^2+^-dependent activation. We used cryoEM to image two GoF hTMEM16E mutants, G503E and R582I ^18, 21, 24^, associated with GDD to elucidate how they affect the structure-activity relationship. Unexpectedly, we found that despite having similar functional and pathological phenotypes, the two mutations act in mechanistically distinct manners: whereas G503E disrupts the TM3-TM4 interface, R582I impairs the arrangement of the TM5-TM6 extracellular loop and increases occupancy of the orthosteric Ca^2+^ binding sites. Using adaptive ensemble molecular dynamics simulations (aeMD) we found that the Ca^2+^-bound closed groove of wild type (WT) hTMEM16E is stable and does not mediate lipid scrambling. In contrast, the G503E mutant readily transitions to a state with an X-shaped groove, similar to that seen in TMEM16F ^49, 50^, that mediates robust lipid scrambling in coarse grained MD simulations. In sum, our results elucidate the basis of physiological and pathological hTMEM16E activation and rationalize its dual-function activity, paving the way for the future development of targeted therapeutic interventions.

## Results

### Purified hTMEM16E is a Ca^2+^ dependent scramblase

**Figure 1:**
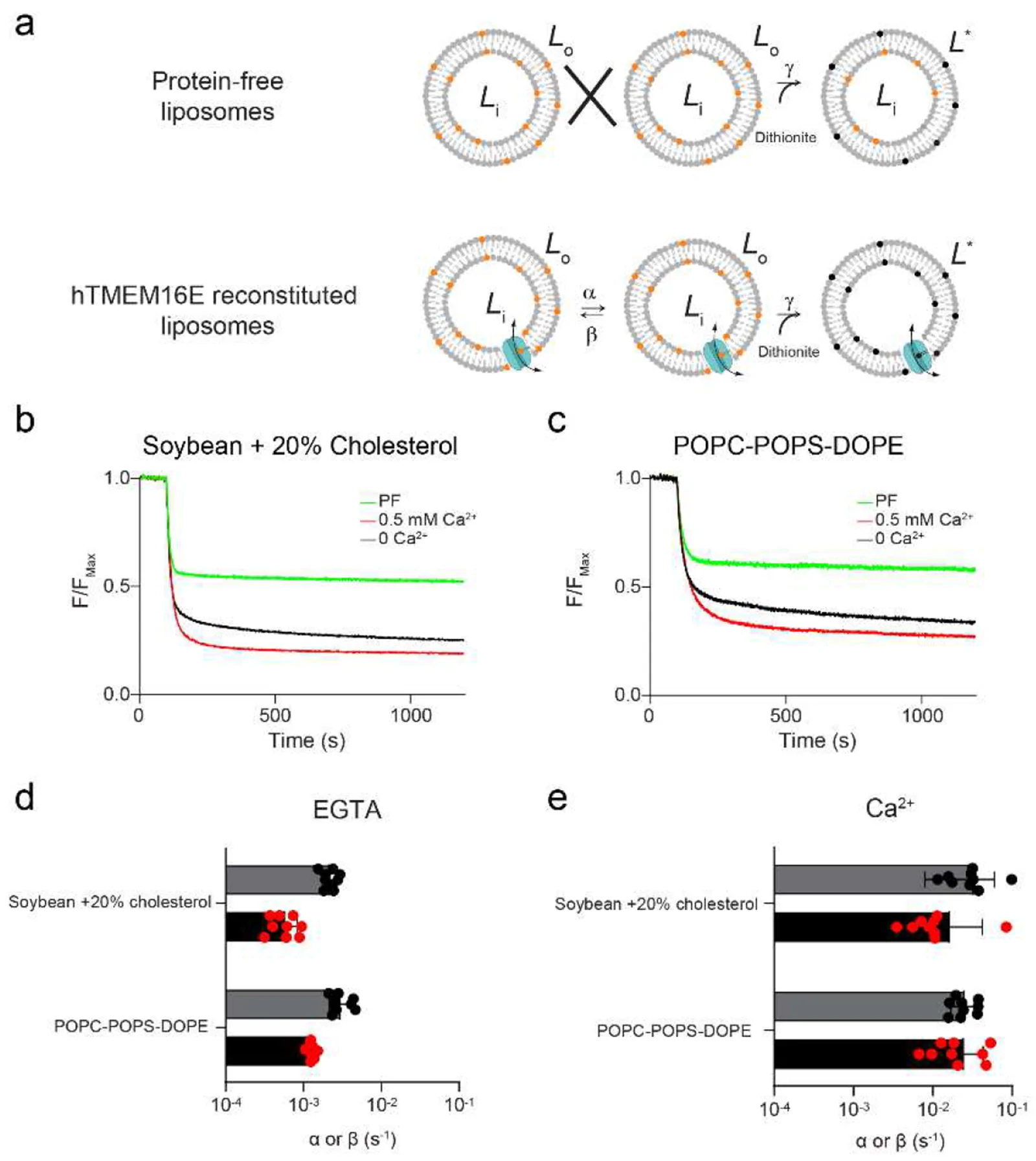
Functional characterization of hTMEM16E. **a** Schematic representation of the dithionite-based in vitro scrambling assay. In protein free (PF) liposomes (top) only outer leaflet NBD-fluorophores (orange) are reduced to non-fluorescent (black) by dithionite. In hTMEM16E-containing proteoliposomes (bottom) NBD-labeled lipids are translocated to the outer leaflet and thus all fluorophores are reduced. **b,c** Representative traces of the dithionite induced fluorescence decay in the scrambling assay for PF liposomes (green), and TMEM16E proteoliposomes in 0 (black) or 0.5 mM Ca^2+^ (red) reconstituted in Soybean polar lipids supplemented with 20% cholesterol (b) or DOPE:POPC:POPS in a 2:1:1 ratio (c) mixed membranes. **d,e** Forward (^, black) and reverse (^, gray) scrambling rate constants of TMEM16E reconstituted in proteoliposomes with NBD-labeled PE lipids in 0 (**d**) or 0.5 mM Ca^2+^ (**e**). Mean ± S. Dev. (n = 9 and N = 3) , red and black circles are values from individual repeats.

We purified hTMEM16E, reconstituted it in proteoliposomes and used an in vitro lipid scrambling assay (Fig. 1a) ^41, 53, 54^ to determine whether it has intrinsic calcium dependent phospholipid scramblase activity, as proposed ^19, 21^. Liposomes were prepared from soybean polar lipids supplemented with 20% cholesterol or from a mixture of 1,2-dioleoyl-sn-glycero-3-phosphoethanolamine (DOPE), 1-palmitoyl-2-oleoyl-sn-glycero-3-phospho-L-serine (POPS) and 1-palmitoyl-2-oleoyl-glycero-3-phosphocholine (POPC) in a 2:1:1 molar ratio. In both cases, 0.4% nitrobenzoxadiazole (NBD)-labeled PE was added to the lipid mix. Addition of the membrane-impermeant reducing agent dithionite irreversibly bleaches only NBD fluorophores in the external leaflet. In protein-free liposomes, this results in ~50% loss of fluorescence (Fig. 1b,c, green trace), whereas in proteoliposomes containing an active scramblase a larger fluorescence loss is expected as NBD-lipids can move between leaflets (Fig. 1b,c). As expected, in hTMEM16E proteoliposomes formed from both lipid mixtures we observe a marked fluorescence reduction (Fig. 1b,c, red and black traces) with similar macroscopic scrambling rate constants (Fig. 1d,e). Interestingly, hTMEM16E has a high basal activity in 0 Ca^2+^ (Fig. 1b,c, black traces), more similar to fungal TMEM16s ^53^ than to its closest mammalian homologue TMEM16F ^50^. Finally, saturating Ca^2+^ elicits only a modest ~10-fold increase in the scrambling rate constants, from ~3x10^−3^ s^−1^ in 0 Ca^2+^ (Fig. 1d) to ~3x10^−2^ s^−1^ in 0.5 mM Ca^2+^ (Fig. 1e), whereas Ca^2+^ elicits a >200-fold increase in other TMEM16s under similar conditions ^39, 41, 50, 53, 55^. Thus, purified hTMEM16E is a Ca^2+^-regulated phospholipid scramblase. Its basal activity and low Ca^2+^ modulation are consistent with its localization to the ER, where constitutively active scramblases help redistribute newly synthesized lipids between leaflets ^56^.

### Cryo-EM structures of Ca^2+^-bound and Ca^2+^-free hTMEM16E

**Figure 2:**
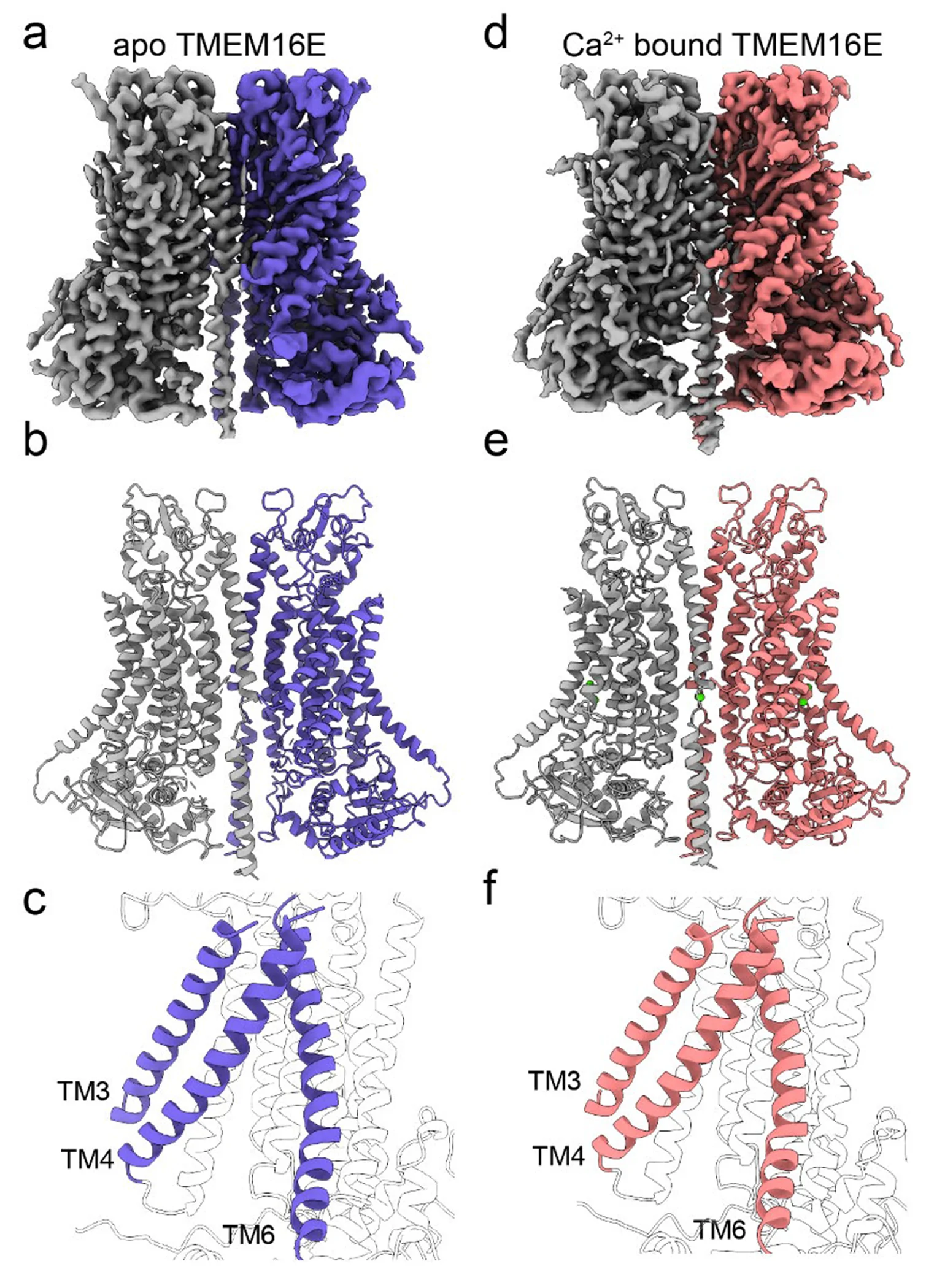
Structure of hTMEM16E **a-c**, CryoEM density (**a**), atomic model (**b**), and close up view of the groove-lining helices TM3-TM6 (**c**) of apo hTMEM16E. One protomer is in gray and the other in blue. **d-f,** same as in (**a-c**) for Ca^2+^ bound hTMEM16E. One protomer is in gray and the other in pink. Ca^2+^ ions in (**e**) are shown as green spheres.

To gain insight into the structural basis of Ca^2+^-dependent hTMEM16E activity, we used single-particle cryo-electron microscopy (Cryo-EM) to determine its structures in the apo and Ca^2+^-bound states. In each condition we identified a single major conformation, and the respective reconstructions reached similar average resolutions, ~3.0 Å in presence of Ca^2+^ and ~3.2 Å in absence of Ca^2+^ (Fig. 2a,b,d,e; Supplementary Fig. 2, 3, 4a; Table 1). As expected, hTMEM16E adopts the canonical dimeric “butterfly” architecture of TMEM16 proteins, where each protomer contains three binding sites for Ca^2+^, a cytosolic one near the dimer interface (allosteric site, S3) and two (orthosteric sites, S1 and S2) near the hydrophilic groove (Fig. 2e). In both conformations the groove is closed by the tight juxtaposition of TM4 and TM6 (Fig. 2c,f) and we observe lipid-like densities near the dimer interface cavity formed by TM3, TM5, TM9, and TM10, possibly due to co-purified lipids (Supplementary Fig. 4a). We also identified extra densities at residues N351, N753, and N763 (Supplementary. Fig. 4b), which could correspond to N-linked glycans, consistent with the idea that TMEM16E primarily localizes to the endoplasmic reticulum (ER), where the first step of the N-glycosylation process occurs ^57, 58, 59^.

**Figure 3:**
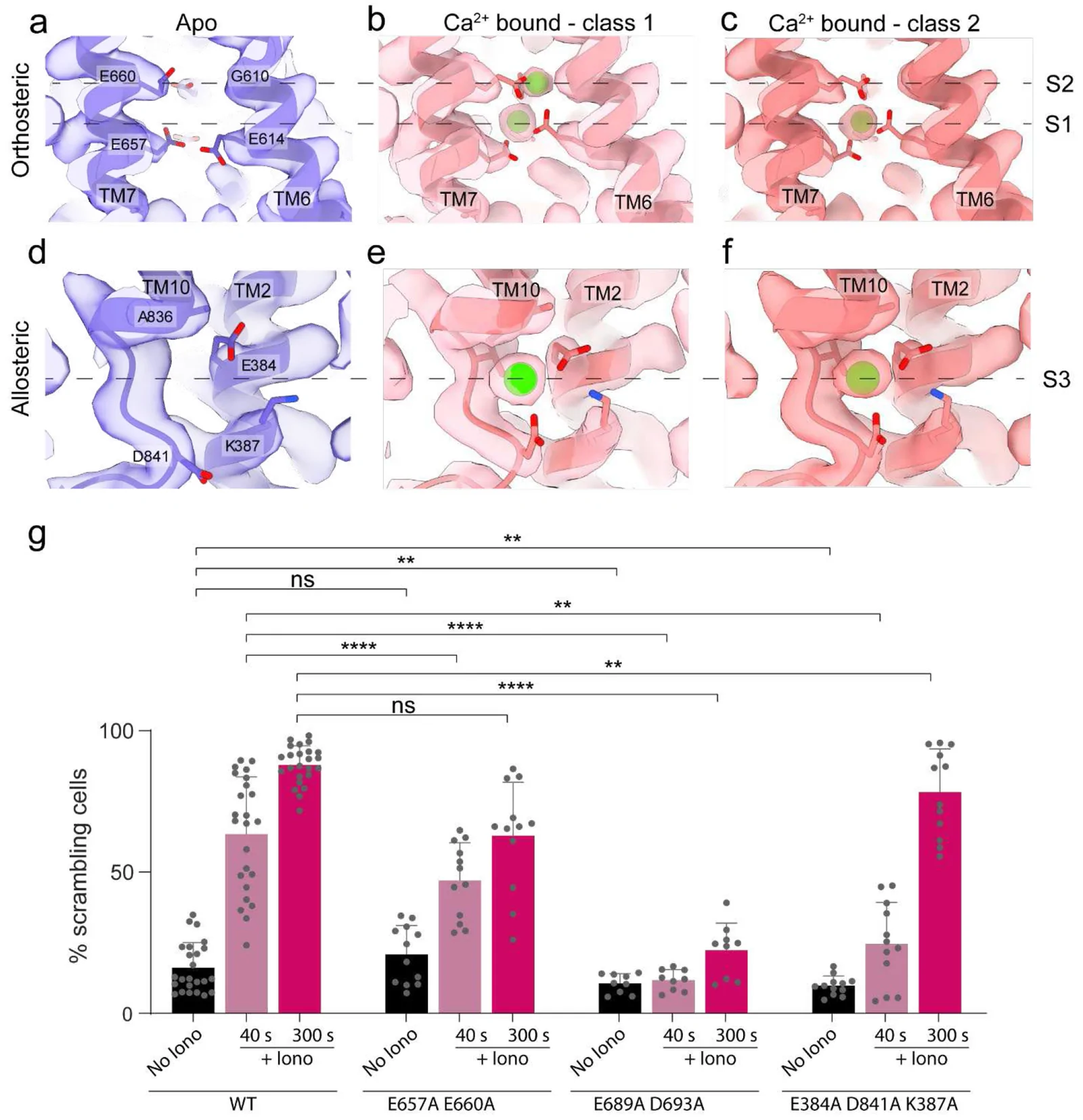
Structure and functional roles of the orthosteric and allosteric Ca^2+-^binding sites in hTMEM16E. **a-f** Close-up views of the cryoEM density and atomic models of the orthosteric S1 and S2 (**a-c**) or the allosteric S3 (**d-f**) Ca^2+^-binding sites in the apo (**a,d**) or Ca^2+^-bound (**b,c,e,f**) states. Side chains of the Ca^2+^ coordinating residues are shown as sticks and the Ca^2+^ ions are displayed as green spheres. The density maps (contoured at 4.0 σ) are shown in purple (apo), light pink (Ca^2+^-bound, Class 1) or coral (Ca^2+^-bound, Class 2). **g** Fraction of scrambling HEK293T TMEM16F KO cells transfected with hTMEM16E WT or Ca^2+^ binding site mutants (E657A/E660A, E689A/D693A, E384A/D841/K387A) in the absence (black bars) or 40 s (light pink bars) or 300 s (dark pink bars) after addition of ionomycin. DAPI-negative, GFP-positive cells were gated and analyzed for Annexin V signal. Bars indicate Mean ± S. Dev. and gray circles are values from individual repeats. The averages are determined from WT (n = 21 repeats and N = 7 independent preparations), E657A/E660A (n = 12 and N = 4), E689A/D693A (n = 9 and N = 3) and E384A/D841/K387A (n = 12 and N = 4). The statistical significance of the effects of the mutants on the scrambling activity was evaluated with an unpaired two-sided t-test with Welch’s correction. ** denotes *P* < 0.01 (P = 0.00698 for WT versus E689A/D693A, *P* = 0.00199 for WT versus E384A/D841/K387A, *P* = 0.001108 and *P* = 0.0011136 for WT versus E657A/E660A at 40s and 300s respectively ), **** denotes P < 10 ^−4^ (*P* < 0.00000001 for WT versus E689A/D693A at 40s and 300s and *P* = 0.00000007 for WT versus E384A/D841/K387A at 40s). ns indicates P > 0.05 and not significant.

The hTMEM16E structures present several unexpected differences compared to those of other family members. First, Ca^2+^ binding fails to elicit any conformational rearrangements in the transmembrane domain: apo and Ca^2+^-bound hTMEM16E are nearly identical, with a Cα r.m.s.d. of ~1.0 Å (Supplementary Fig.4c-e). Ca^2+^ binding induces only a slight rotation of TM10b and of the ferredoxin domain (Supplementary Fig. 4c). This is unlike other TMEM16s, where in the absence of Ca^2+^ TM6 is bent and the orthosteric binding sites are disrupted, and Ca^2+^ binding induces extensive movements of the cytosolic domain, a straightening of TM6 and, often, a displacement of TM4 (Supplementary Fig. 4f-i) ^39, 43, 44, 45, 46, 47, 60^. In contrast, in apo hTMEM16E, TM6 is straight (Supplementary Fig. 4d-e) and the side chains forming the orthosteric sites are properly positioned, even though the sites are empty (Fig. 3a). Second, in saturating 1 mM Ca^2+^ the two orthosteric binding sites are not fully occupied as the density corresponding to the Ca^2+^ ion in the S2 site is much weaker than that in the S1 or S3 sites (Fig. 3b,c,e,f), suggesting partial occupancy of the S2 site (Fig. 3b,c). To evaluate occupancy in individual protomers we carried out symmetry expansion and signal subtraction and identified two distinct classes (Supplementary Fig. 3e): class 1 where all three sites are occupied, but the S2 site is considerably weaker than the other two, and class 2 with only the S1 and S3 sites occupied (Fig.3 b,c,e,f). Third, in Ca^2+^-bound hTMEM16E the tilt of TM4 relative to the membrane plane is more pronounced compared to that seen in Ca^2+^-bound closed TMEM16F in digitonin (PDB 6QP6), in a pose more like that seen in liposomes (PDB 9O1M) (Supplementary Fig. 4h,i). Finally, alignment of the apo and Ca^2+^ bound structures of TMEM16E and TMEM16F shows that TM6 and the cytosolic ferredoxin domain are the main regions of structural divergence (Supplementary Fig. 4f-i) ^44, 50^.

### Dissecting the role of the Ca^2+^ binding sites in TMEM16E function

Our data show that hTMEM16E activity is only modestly Ca^2+^ dependent (Fig. 1d,e), that Ca^2+^ binding fails to elicit major conformational rearrangements in the transmembrane region (Supplementary Fig. 4d), and that the S2 orthosteric site is not fully occupied even in 1 mM Ca^2+^ (Fig.3 b,c,e,f). In contrast, other TMEM16s display stronger Ca^2+^-dependence, undergo Ca^2+^-dependent rearrangements and both orthosteric sites are occupied ^39, 43, 44, 46, 60^. These structural and functional differences raise the question of the precise roles played by the three sites in hTMEM16E activation. To dissect the role of the orthosteric and allosteric sites we disrupted the S1 and S2 sites by mutating to alanine conserved residues on TM7 (E657, E660) or TM8 (E689, D693) and the S3 site by mutating conserved residues on TM2 and TM10 (E384, K387, D841) (Supplementary. Fig. 5a,b). We used a cell-based flow cytometry assay to assess the functional impact of mutants on hTMEM16E scrambling activity. In this assay phosphatidylserine (PS) externalization is initiated by the addition of ionomycin, a Ca^2+^ ionophore, and is quantified by monitoring binding of fluorescently labeled Annexin V to the plasma membrane of HEK293 cells where endogenous TMEM16F has been knocked out ^61^ and overexpressing WT or mutant hTMEM16E. The fraction of Annexin V–positive cells was quantified at 40 s and 300 s. As expected, WT and mutant hTMEM16E show minimal scrambling in the absence of ionomycin (Fig. 3g black bar, Supplementary Fig. 5c). In cells transfected with WT hTMEM16E, elevation of intracellular Ca^2+^ results in an increase of the fraction of Annexin V–positive cells to 63±20% after 40 s and to 87±6% at 300 s. The TM7 double mutant E657A/E660A mildly impairs TMEM16E activation, resulting in 47±13% and 62±18% of Annexin-V positive cells at 40 and 300 s (Fig. 3g light and dark pink respectively). In contrast, the E689A/D693A double mutant on TM8 nearly abolished scrambling, with only 22±9% positive cells at 300 s (Fig. 3g dark pink). Disruption of the S3 allosteric site via the triple E384A/K387A/D841A mutant also impaired scrambling, reducing the fraction of Annexin V–positive cells to 24±14% and 78±15% at 40 and 300 s, respectively (Fig. 3g light and dark pink respectively).

### Structural rearrangements caused by GDD mutations

**Figure 4:**
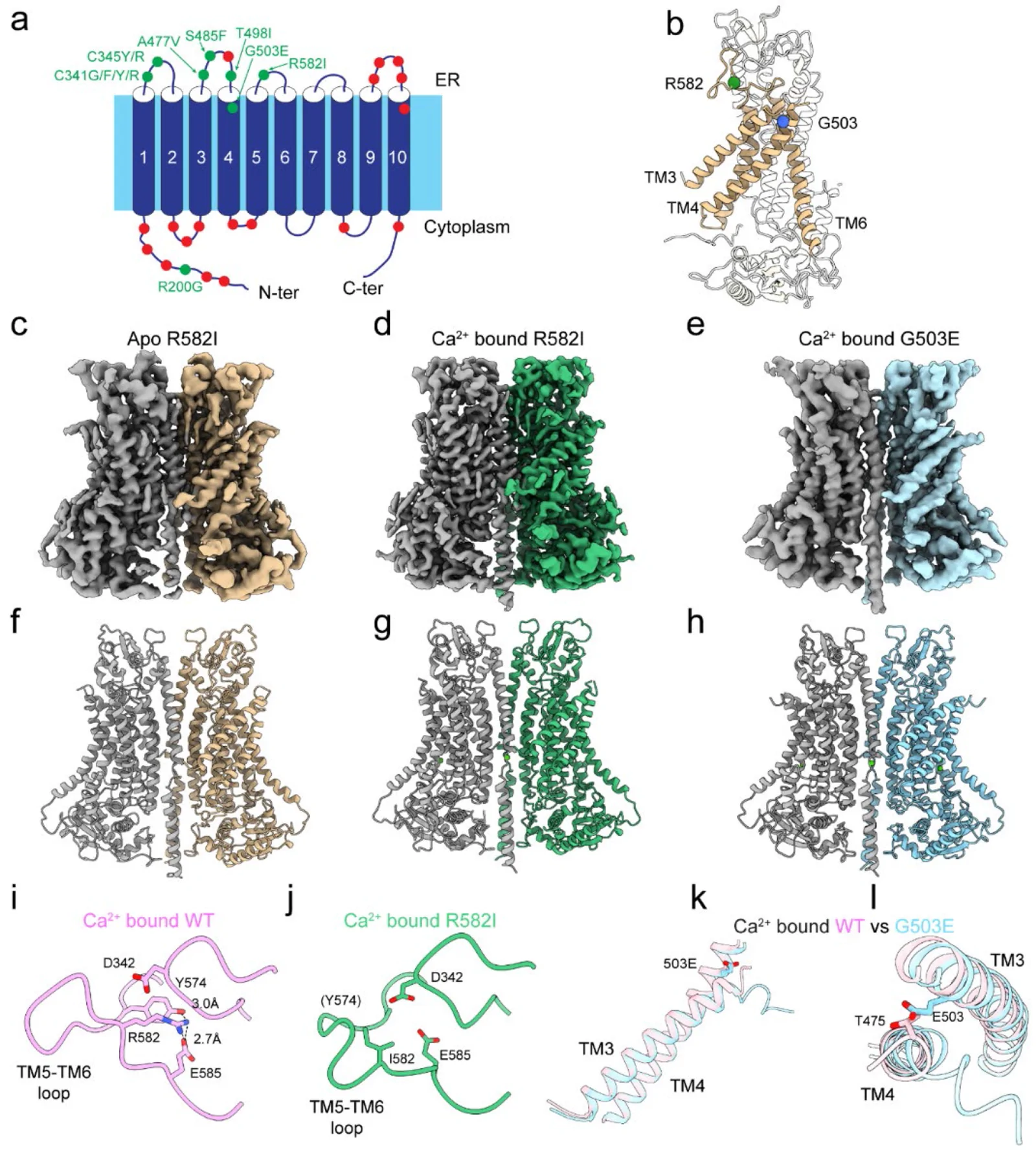
Structural consequences of pathogenic GDD mutations. **a** hTMEM16E topology indicating the position of pathogenic muscular dystrophy (red) and GDD (green) mutations. GDD mutations shown in the panel: R200G, C341G/F/Y/R, C345Y/R, A477V, S485F, T498I, G503E, R582I. Locations of representative MD-associated missense mutations shown in this panel: R43W, G216V, P251L, R389L, S491G, R532Q, S540I, F563S, T684S, R743C, C789S, A815K, M818K, and M885L. Residue numbering corresponds to the 898-aa hTMEM16E isoform. **b** The R582I (green) and G503E (light blue) GDD mutations are mapped on the structure of WT hTMEM16E. **c-h** CryoEM density maps (**c-e**) and ribbon representation of the atomic models (**f-h**) of apo (**c,f**) and Ca^2+^-bound (**d,g**) R582I and Ca^2+^-bound G503E (**e,h**) hTMEM16E at 3.5, 3.2 and 4.1 Å, respectively. One protomer is shown in gray and the other in sand (apo R582I), green (Ca^2+^-bound R582I), and cyan (Ca^2+^-bound G503E). Ca^2+^ ions in g,h are shown as green spheres. **i,j** Close up view of the TM5-TM6 extracellular loop shown in ribbon representation of Ca^2+^-bound WT (**i**, light pink) or R582I (**j**, green) hTMEM16E. Relevant side chains are shown in stick representation and labeled. Parentheses indicate side chains for which the cryoEM density was of insufficient quality for placement. **k,l** Structural rearrangement of the TM3–TM4 region induced by the GDD-causing G503E substitution. Side (**k**) and top (**l**) views of the superposed TM3 and TM4 helices in Ca^2+^-bound WT and G503E hTMEM16E. The G503 and T475 side chains are shown in stick representation.

Mapping the position of disease-causing mutations on the hTMEM16E structure shows that whereas muscular dystrophy mutations primarily cluster to the N-terminal ferredoxin domain, GDD substitutions predominantly localize to extracellular loops or in groove-lining helices (Fig. 4a,b). To test whether GDD mutations in the loop or in the helices share a common pathological activation mechanism we focused on two variants: the previously reported G503E, which is located at the extracellular end of TM4 where it interfaces with TM3 ^17, 24^, and the recently identified R582I, which is located in the TM5-TM6 loop where it forms a salt bridge with D342 on the TM1-TM2 loop (Fig. 4 a,b,i) ^18^.

**Figure 5:**
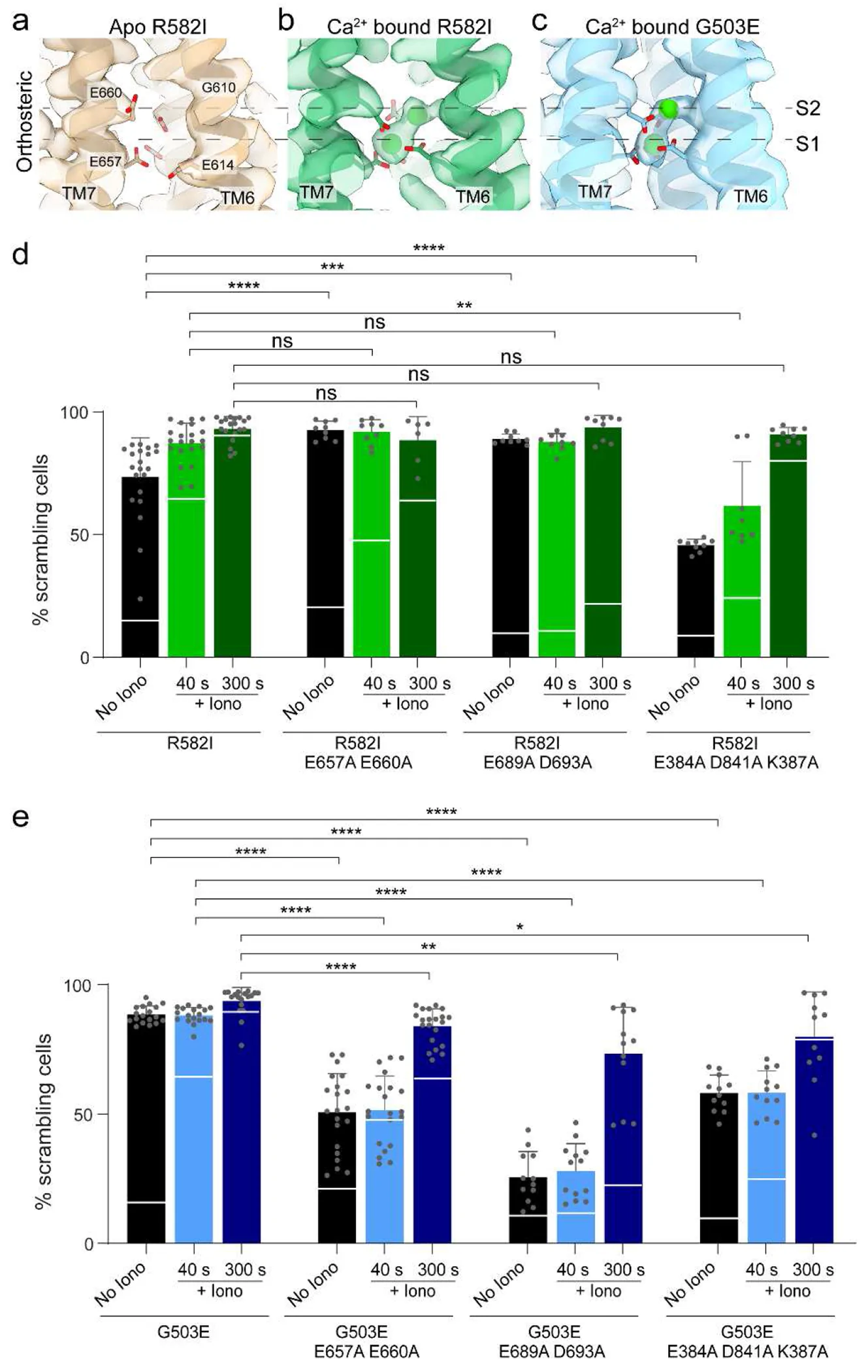
Interplay between Ca^2+-^binding and GDD causing mutations. **a-c** Close-up views of the orthosteric S1 and S2 (**a-c**) Ca^2+^-binding sites in the apo (**a**) and Ca^2+^-bound (**b**) R582I or Ca^2+^-bound G503E (**c**). Side chains of the Ca^2+^ coordinating residues are shown as sticks and the Ca^2+^ ions are displayed as green spheres. The density maps (contoured at 4.0 σ) are shown in wheat (**a**, R582I apo), green (**b**, R582I Ca^2+^-bound) or light blue (**c**, G503E Ca^2+^-bound). **d,e** Fraction of scrambling HEK293T TMEM16F KO cells transfected with R582I (**d**) or G503E (**e**) TMEM16E mutants alone or in combination with Ca^2+^ binding site mutants (E657A/E660A, E689A/D693A, E384A/D841/K387A) in the absence (black bars) or 40 s (light green bars (**d**) or light blue bars (**e**)) or 300 s (dark green bars (**d**) or dark blue bars (**e**)) after addition of ionomycin. DAPI-negative, GFP-positive cells were gated and analyzed for Annexin V signal. Bars indicate Mean ± S. Dev. and gray circles are values from individual repeats. Horizontal white lines superimposed to the colored bars indicate the corresponding values for WT hTMEM16E (taken from Figure 3g). The averages are determined from R582I (n = 21 repeats and N = 7 independent preparations), R582I/E657A/E660A (n = 9 and N = 3), R582I/E689A/D693A (n = 9 and N = 3), R582I/E384A/D841/K387A (n = 9 and N = 3), G503E (n = 18 repeats and N = 6 independent preparations), G503E/E657A/E660A (n = 21 and N = 7), G503E/E689A/D693A (n = 12 and N = 4) and G503E/E384A/D841/K387A (n = 12 and N = 4). The statistical significance of the effects of the mutants on the scrambling activity was evaluated with an unpaired two-sided t-test with Welch’s correction. * denotes *P* < 0.05 (*P* = 0.0261728 for G503E versus G503E/E384A/D841/K387A at 300 s). ** denotes *P* < 0.01 ( *P* = 0.00467972 for R582I versus R582I/E384A/D841/K387A at 40 s and *P*= 0.00239429 for G503E versus G503E/E689A/D693A at 300 s). *** denotes P < 10^−3^ (*P* = 0.00025755 for R582I versus R582I/E689A/D693A), **** denotes P < 10 ^−4^ (*P* < 0.00000001 for R582I versus R582I/E384A/D841/K387A in absence of ionomycin, for G503E versus G503E/E657A/E660A, G503E/E689A/D693A and G503E/E384A/D841A/K387A both in absence of ionomycin and after ionomycin addition at 40 s, and *P*= 0.0000140 for G503E versus G503E/E657A/E660A at 300 s).

We used cryoEM to image these mutants to understand how they affect hTMEM16E activation. We imaged R582I in 0 and 1 mM Ca^2+^ and G503E in 1 mM Ca^2+^ (Fig. 4c–h, Supplementary Fig. 6-7; Table 1). Apo and Ca^2+^-bound R582I are overall similar to their respective WT counterparts with Cα r.m.s.d. of 0.9 and 1.3 Å (Fig.4c,d,f,g; Supplementary Fig 8a,b). In both states the groove is closed, indicating that the mutation does not induce groove opening, at least in our conditions. Rather, the mutation induces a structural rearrangement of the TM5-TM6 loop and increased occupancy of the orthosteric S2 site (Fig. 4i, j; Fig. 5b; Supplementary Fig. 8c,d). In WT hTMEM16E R582 on the TM5-6 loop is wedged in a hydrophilic pocket lined by D342, Y574, and E585, which stabilizes the interactions with the TM1-TM2 extracellular loop via salt-bridge and cation- π interactions (Fig. 4i). In contrast, in hTMEM16E R582I this network of interactions is disrupted, as the TM5-6 loop rearranges and the hydrophobic 582I side chain moves out of the hydrophilic pocket and points towards the membrane (Fig.4j), thus disrupting the organization of the extracellular loops. The second, and unexpected, effect of the R582I mutation is that we observe an apparent strengthening of Ca^2+^ binding to the S2 orthosteric site (Fig. 5b). Indeed, extensive classification of the mutant dataset in Ca^2+^ yielded a single class where both the S1 and S2 orthosteric sites are occupied, although the peak intensity of the S2 site is weaker than that of S1 (Supplementary Fig. 8c,d). This is in contrast to the WT case where we could separate two classes, one of which had an empty S2 site (Fig.3 b,c, Supplementary Fig. 3). This suggests that the R582I mutant disrupts the packing of the extracellular loops of hTMEM16E and strengthens Ca^2+^ binding to the S2 site, which could rationalize its GoF phenotype.

The structure of Ca^2+^-bound G503E shows that the mutation disrupts the extracellular TM3-TM4 interface, resulting in a kink of the TM3 and a slight movement of TM4 (Fig. 4e,h; Supplementary Fig. 7). In WT hTMEM16E, the side chain of T475 on TM3 sits in a small pocket formed by G503 on TM4 (Fig. 4l), stabilizing the TM3-TM4 interface. The introduction of a bulkier and negatively charged glutamate side chain in G503E induces a steric clash with T475 resulting in slight displacement of TM4, which slides along TM6 away from the groove by ~1 Å (Fig. 4l). Additionally, TM3 rearranges to accommodate the acidic E503 side chain, by bending ~7.5° starting at A474 (Supplementary Fig.8e), followed by a loss of helical structure at A474 which results in a partial unwinding and kinking (Fig. 4l, Supplementary. Fig. 8e). These rearrangements are reminiscent of those seen in F518H mTMEM16F in detergent ^49^, although in that structure TM4 fully disengaged from TM3 which had a more pronounced kink (Supplementary Fig. 8f). Notably, the observed repositioning of TM4 in G503E hTMEM16E is closer to that seen in the F518H mTMEM16F in nanodisc, which likely represents an intermediate along the activation to the X-shaped groove ^49^. These findings suggest that G503E favors activation by disrupting the TM3-TM4 interface.

We tested this hypothesis using the flow cytometry assay to investigate the functional effects of the G503E and R582I variants alone and in combination with mutations disrupting the orthosteric and allosteric Ca^2+^-binding sites. We found that the G503E and R582I mutants induce constitutive PS exposure in 88.4±3.3% and 73±15% of cells even in the absence of ionomycin treatment (Fig. 5d,e black bars; Supplementary Fig. 9), ~5-fold higher than that seen in WT-expressing cells (Fig. 3g black bars). This fraction increased slightly to 87±8% upon increase in intracellular Ca^2+^ in R582I (Fig. 5d light and dark green bars). Thus, both variants have similar functional effects and induce constitutive scramblase activity at basal cellular Ca^2+^ levels, consistent with their GDD disease phenotype. Then we investigated the combined effects of these GoF mutations with the LoF mutations at the Ca^2+^ binding sites. Consistent with our structural results, the R582I mutant rescues the disruption of scrambling activity caused by mutations at the orthosteric sites, whereas mutating the allosteric site resulted in a modest impairment in activation (Fig. 5d). In contrast, the disruptive effects of mutations at the orthosteric and allosteric Ca^2+^ binding sites are retained on the background of the G503E mutant (Fig. 5e). Together these results support the idea that the two GoF mutations, both associated with a similar GDD disease phenotype, act via different mechanisms: the R582I mutant increases Ca^2+^ binding to the orthosteric site whereas the G503E mutant disrupts the TM3-TM4 interface.

### The G503E mutation favors transition to an active X-shaped groove

**Figure 6:**
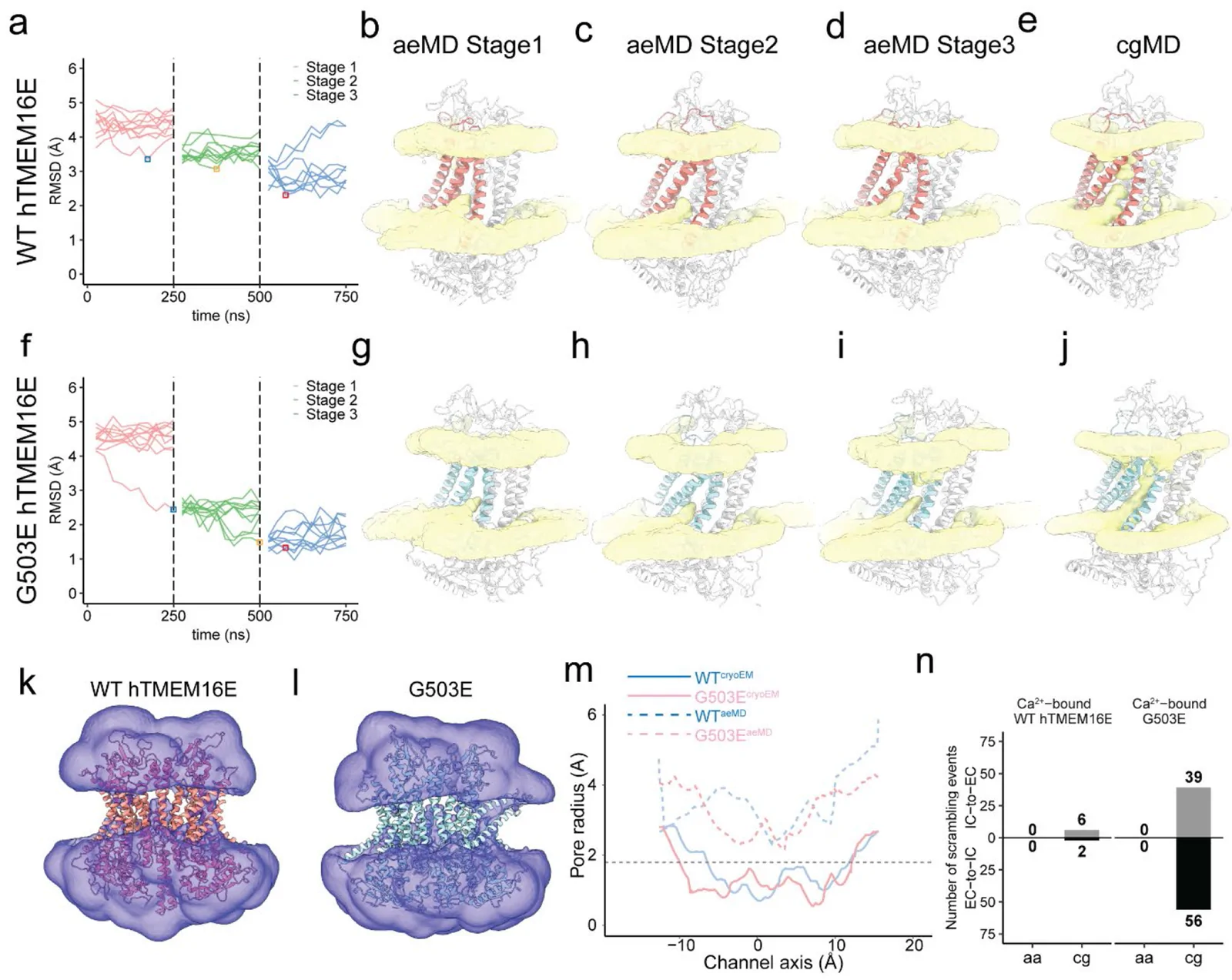
Activation mechanism and basis of lipid scrambling by hTMEM16E. **a,f** Time course of the Cα R.M.S.D. of groove helices TM4-TM7 in protomer A to hTMEM16E^Act^ during adaptive ensemble MD simulation trajectories of WT (**a**) and G503E (**f**) hTMEM16E. Individual traces correspond to each of 10 independent trajectories each 250 ns long. Blue/orange/red squares indicate the frame used to start the Stage 2, 3 and cgMD simulation runs, respectively. **b-e, g-j,** The average density of the lipid phosphate atoms in aeMD (**b-d** and **g-i**) or of the PO4 bead in cgMD (**e,j**) simulations within 10 Å of the groove of WT (**b-e**) and G503E (**g-j**) in stage 1 (**b,g**) stage 2 (**c,h**), stage 3 (**d,i**), and cgMD (**e,j**). The density is shown in a transparent yellow surface at a 2.5% occupancy threshold. The atomic models of the proteins are shown in ribbon representation and correspond to the cryoEM structures for stage 1, and to the frames indicated by the squares for the subsequent ones. **k-l,** The average density of the water molecules in stage 3 aeMD simulations within 10 Å of WT (**k**) and G503E (**l**). The density is shown in a transparent blue surface at a 10% occupancy threshold. The atomic models of the proteins are shown in ribbon representation and correspond to the frames indicated by the red squares in panels **a** and **f**, respectively. **m,** The internal pore radius of the groove of WT (red lines) and G503E (blue lines) hTMEM16E in the cryoEM (solid lines) or stage 3 aeMD conformations (dashed lines). Dashed gray line indicates 1.8 Å which is the radius of the Cl^−^ ion. **n,** Total number of lipid scrambling events at the groove seen in Stage 3 aaMD and in cgMD simulations of Ca^2+^-bound WT and G503E TMEM16E from Intracellular (IC) to Extracellular (EC) (gray bars) or from EC to IC (black bars).

We used molecular dynamics (MD) simulations to gain insights into the scrambling mechanism of hTMEM16E and to understand how the G503E mutation favors activation. First, we performed all-atom MD (aaMD) simulations to test whether Ca^2+^-bound WT or G503E hTMEM16E scramble lipids. For both constructs 10 independent trajectories, each 250 ns long, were run. In both cases we only observed minor membrane thinning near the groove (Fig. 6b,g) and no scrambling events (Fig. 6n), supporting the notion that the closed-groove state is inactive. Notably, whereas in all WT trajectories we observe only minimal structural rearrangements (Fig. 6a, Supplementary Fig. 10a, d-e), in one of the G503E trajectories we observed a spontaneous rearrangement in one protomer where TM4 separates from TM3 and slides along TM6 (Fig. 6f, Supplementary Fig. 10b,h-i), resulting in a groove that begins to resemble that of an homology model of active hTMEM16E, hTMEM16E^Act^, generated using SwissModel ^62^ based on the X-shaped active state of TMEM16F (PDBID: 9O1N) (Supplementary Fig. 10c). Thus, we hypothesized the observed transition could represent a step towards activation. We tested this hypothesis in a 3-stage adaptive ensemble MD (aeMD) for WT and G503E hTMEM16E using as target hTMEM16E^Act^ and the initial aaMD trajectories as stage 1. As initial frames for stage 2 and 3 simulations we chose frames with the lowest Cα r.m.s.d. of the groove-lining TM4-TM7 helices to target in the preceding stage (Fig. 6a,f). The aeMD protocol was applied only to protomer A, while protomer B evolution was unbiased, as a control. Each stage comprised of 10 independent trajectories each 250 ns long. In stage 1, the initial Cα r.m.s.d. between the cryoEM structures and hTMEM16E^Act^ is ~4.4 and 4.2 Å for WT and G503E hTMEM16E, respectively (Fig. 6a,f). Over the course of the 3 stages of aeMD, protomer A of WT hTMEM16E transitions to a conformation, WT^aeMD^, where TM4 undergoes a slight rearrangement away from TM3, such that the average stage 3 Cα r.m.s.d. to the active state decreases to ~3.1 Å (Fig. 6a; Supplementary Fig. 10d-g). As expected, protomer B remains largely unchanged (Supplementary Fig. 10a). In WT^aeMD^ the groove remains closed to the membrane (Fig. 6d), the internal pore dilates and becomes hydrated (Fig. 6m), and membrane thinning is modest (Fig. 6c,d). This conformation is not stable, as we observe spontaneous reversion to a fully closed groove in 2 of 10 replicas (Fig. 6a), suggesting it might represent a metastable intermediate. In contrast, the groove in protomer A of G503E progressively and stably transitions to states that resemble the X-shaped state (Fig. 6f; Supplementary Fig. 10h-k), leading to increased membrane thinning (Fig. 6g–i). The resulting conformation, G503E^aeMD^, is nearly indistinguishable from hTMEM16E^Act^, with a stage 3 Cα r.m.s.d. average of ~1.6 Å (Fig. 6f; Supplementary Fig. 10k). Protomer B of G503E remains largely unchanged (Supplementary Fig. 10b). The groove of G503E^aeMD^ forms a wide and continuous transmembrane pore that is hydrated (Fig. 6l) and wide enough to allow ion permeation (Fig. 6m). Notably, in G503E^aeMD^ the extracellular vestibule of the groove becomes pronouncedly more hydrated near the TM3 helix than in WT^aeMD^, likely reflecting the combined effects of the additional charged residue and the disruption of the TM3-TM4 interface (Fig. 4k–l; Supplementary Fig. 8e).

During these atomistic simulations we did not observe any full scrambling events (Fig. 6n), despite observing a conformation-dependent increase in membrane thinning in both WT and G503E hTMEM16E (Fig. 6b–d, g–i). To enhance sampling and evaluate the lipid scrambling competence of WT^aeMD^ and G503E^aeMD^ we ran 10 independent replicas each 5 μs long of coarse-grained MD (cgMD) simulations. In cgMD simulations of WT^aeMD^ we observed pronounced membrane thinning at the groove (Fig. 6e) and 137 lipid scrambling events, of which 8 occurred at the TM4-TM6 groove in Protomer A. The other events occurred at the dimer cavity or in other regions at the periphery of the protein. This is consistent with recent reports for cgMD simulations of TMEM16 homologues using the Martini3 forcefield ^50, 63^. These events are likely not mechanistically relevant, and therefore they were excluded from our analysis. In cgMD simulations of G503E^aeMD^ we observe pronounced membrane thinning near the groove (Fig. 6j) which is accompanied by 95 events occurring at the X-shaped groove of protomer A of G503E^aeMD^ (Fig. 6n). We also observed 73 events in other regions. Together, these results support our hypothesis that hTMEM16E adopts an active conformation that resembles the X-shaped groove of TMEM16F ^50^, and that the G503E mutations favors activation by disrupting the TM3-TM4 interface. Further, our data suggests that, like TMEM16F, hTMEM16E scrambles lipids outside an X-shaped groove.

## Discussion

The human TMEM16E protein is a Ca^2+^ activated channel and scramblase and plays a vital role in muscle and bone homeostasis. Its dysfunction is associated with inherited disorders such as autosomal recessive limb-girdle muscular dystrophy-12, Miyoshi muscular dystrophy-3 ^28, 29^, and dominant gnathodiaphyseal dysplasia 1 ^13, 14^. Despite its importance in human pathophysiology there is no information on the structural basis of hTMEM16E activation or how disease-causing mutations affect this process. Further, recent work suggests that activation of TMEM16 family members can entail different rearrangements: in the ER-resident TMEM16K, and in fungal nhTMEM16 and afTMEM16 the lipid groove opens and forms a shared pathway for ions and lipids ^39, 42, 43, 60^. In contrast, in the PM-resident TMEM16F the groove adopts an X-shaped conformation that forms a transmembrane pore to conduct ions and thins the membrane to allow lipid scrambling along its periphery^50^. To understand how Ca^2+^ activates hTMEM16E, we determined the cryoEM structures of apo and Ca^2+^-bound hTMEM16E (Fig. 2). Unexpectedly, we found that despite high sequence and structural homology to other TMEM16 family members, hTMEM16E presents several unique structural and mechanistic features. First, Ca^2+^ binding induces nearly no conformational changes in the transmembrane region of the protein since in both apo and Ca^2+^-bound hTMEM16E TM6 is straight, the orthosteric sites are formed, and the lipid groove is closed (Fig. 2). Further, even in saturating Ca^2+^ the S2 orthosteric site is partially unoccupied (Fig. 3). Consistently, TMEM16E displays weaker Ca^2+^ dependence than other family members (Fig. 1) ^39, 41, 42, 44, 64^, and mutations disrupting the orthosteric sites do not completely ablate its Ca^2+^-dependent activation (Fig. 3).

Notably, we found that two GDD mutations, G503E and R582I, which have similar functional and pathological phenotypes, act via fundamentally distinct mechanisms: whereas G503E disrupts the TM3-TM4 interface to facilitate activation, R582I increases occupancy of the S2 orthosteric Ca^2+^ binding site, likely an allosteric effect of the disruption of the TM5-TM6 extracellular loop. Using MD simulations we found that the G503E mutant favors transition to a state with an X-shaped groove that mediates robust lipid scrambling in cgMD. This conformation is similar to the active state of TMEM16F ^49, 50^, suggesting a common activation mechanism of these two homologues. These findings underscore the mechanistic diversity within the TMEM16 family and suggest that the cellular localization of TMEM16 homologues does not correlate with their activation mechanism. Indeed, while TMEM16E and TMEM16K are Ca^2+^-dependent dual function scramblases and channels that primarily localize to the ER, they adopt different active states: TMEM16K adopts an open-groove conformation reminiscent of that seen in fungal afTMEM16 and nhTMEM16 ^39, 43, 60^ while TMEM16E scrambles lipids outside an X-shaped groove that is similar to that of the PM-localized TMEM16F ^49, 50^ (Fig. 6).

Our findings shed some light onto the thus far inscrutable role of the extracellular loops in TMEM16E pathophysiology. While these loops are recognized as disease hotspots (Fig. 4), harboring causative mutations for LGMDR12, MMD3 and GDD, such as C341 or T498 which are among the most frequently mutated positions in GDD, their role was unknown. Notably, the effects of mutations in these loops are conserved across TMEM16 homologues ^65^, suggesting a conserved functional role. Our structures show that in WT hTMEM16E the precise arrangement of the TM1-TM2 and TM5-TM6 loops is controlled by an evolutionarily conserved network of interactions involving R582 and D342 as well as the disulfide bond linking C341 and C345 (Fig. 4i,j). The GDD-causing R582I mutant disrupts these interactions, leading to the repositioning of the TM5-TM6 loop away from the TM1-TM2 loop (Fig. 4i,j, Supplementary Fig. 5d). The altered loop arrangement is associated with increased Ca^2+^ occupancy of the orthosteric S2 site, which favors activation of hTMEM16E. We speculate that other disease mutations in these loops, such as those disrupting the C341-C345 disulfide, are also likely to disrupt the arrangement of these loops and thus exert their pathogenic effects via a similar allosteric mechanism.

In summary, our findings provide a framework to begin unraveling the relationship between hTMEM16E structure, function, and disease. Our results revealed that hTMEM16E has several distinctive features. Importantly, we found that GoF mutations are phenotypically similar and yet act via distinct structural mechanisms, underscoring the need to elucidate the precise molecular mechanisms underlying disease. We expect that our findings will pave the way for the development of mutant-specific approaches aimed at correcting disease phenotypes associated with hTMEM16E dysfunction.

## Supplementary Material

This is a list of supplementary files associated with this preprint. Click to download.

- DiZannietalMethods.pdf
- DiZannietalMethods.pdf
- DiZannietalTable1.pdf
- DiZannietalSI.pdf

Table 1 is available in the Supplementary Files section.

## Data Availability

The data that support this study are available from the corresponding author upon request. All models and associated cryoEM maps have been deposited into the Electron Microscopy Data Bank (EMDB) and the Protein Data Bank (PDB). The depositions include final maps, unsharpened maps, local refined maps, and associated FSC curves. Accession codes are listed here and in Table 1. WT TMEM16E in the absence of Ca^2+^: EMD- 77883 and PDB 36UZ ; Ca^2+^-bound closed WT TMEM16E: EMD- 78009 and PDB 37AM ; Apo R582I TMEM16E: EMD- 78021 and PDB 37AY; Ca^2+^-bound R582I TMEM16E: EMD- 78017 and PDB 37AW ; Ca^2+^ bound G503E TMEM16E: EMD- 78028 and PDB 37BB . Maps, half-maps and atomic models are available for download at: https://figshare.com/s/9d4baf806e623bb602e7 . Representative MD trajectories are available for download at: all atom Ca^2+^ bound WT https://figshare.com/s/c6cbd8c25ec1ef4f1cc9; all atom Ca^2+^ bound G503E https://figshare.com/s/1f384a25fc217b4a357e; coarse grained Ca^2+^ bound WT https://figshare.com/s/34ec2af8aaec35954e24; coarse grained Ca^2+^ bound G503E https://figshare.com/s/fc1037cac369b59f5dc1.

## Code availability

Custom analysis scripts to identify lipid scrambling events in MD simulation trajectories are downloadable at: all atom Ca^2+^ bound WT https://figshare.com/s/c6cbd8c25ec1ef4f1cc9; all atom Ca^2+^ bound G503E https://figshare.com/s/1f384a25fc217b4a357e; coarse grained Ca^2+^ bound WT https://figshare.com/s/34ec2af8aaec35954e24; coarse grained Ca^2+^ bound G503E https://figshare.com/s/fc1037cac369b59f5dc1.
